# Interdisciplinary collaboration in pediatric palliative care: a qualitative study on barriers and facilitators as perceived by parents and healthcare professionals

**DOI:** 10.1007/s00431-026-07070-7

**Published:** 2026-05-20

**Authors:** Maureen M. Kemna, Judith L. Aris-Meijer, A. A. Eduard Verhagen, Saskia C. C. M. Teunissen, Marijanne Engel, Marijke C. Kars

**Affiliations:** 1 Julius Center for Health Sciences and Primary Care, Department of General Practice & Nursing Science, Center of Expertise in Palliative Care Utrecht, University Medical Center Utrecht, Utrecht University, Universiteitsweg 100, 3548 CG Utrecht, The Netherlands; 2 University of Groningen, University Medical Center Groningen, Beatrix Children’s Hospital, Hanzeplein 1, 9713 GZ Groningen, The Netherlands

**Keywords:** Pediatric palliative care, Interdisciplinary collaboration, Facilitators, Barriers, Situational care networks

## Abstract

Pediatric palliative care (PPC) requires involvement of various healthcare professionals (HCPs) across home and hospital settings to address the complex needs of children and families. Interdisciplinary collaboration (IDC) among HCPs and parents is crucial for the coordination, continuation, and quality of PPC. Yet, IDC remains difficult to achieve in practice. We aimed to identify barriers and facilitators to IDC in PPC as experienced by expert parents and HCPs in order to strengthen PPC. An exploratory multiple-case study was conducted using semi-structured interviews. Cases consisted of (non-)bereaved parents of a child qualifying for PPC and their involved HCPs. Data was thematically analyzed using the QUAGOL method. Nine cases, representing nine children, were included, comprising interviews with 14 parents and 39 HCPs. Barriers and facilitators to IDC were context-dependent and spanned seven domains: network, interdependence, goals of care, roles and tasks, added value, responsibility, and urgency. IDC typically developed slowly after diagnosis due to barriers such as negative perceptions on collaborative partners or IDC. Collaboration intensified during crisis and the terminal phase due to facilitators such as awareness of being a network.

*Conclusion*: IDC in PPC cases is best understood as occurring in situational care networks (SCNs): temporary, child-specific constellations of parents and fluctuating non-standard and transmural collaborating HCPs. This conceptualization exposes vulnerabilities, but also opportunities of support and complementary HCP expertise in PPC collaboration. Early initiation of SCNs seems to better strengthen structural IDC than reactive reliance on parental coordination or crisis-driven senses in PPC.

**Table:** 

**What is Known:** • *Interdisciplinary collaboration among healthcare professionals and parents is crucial for coordination, continuation, and quality of pediatric palliative care.* • *Parents and healthcare professionals report challenges regarding collaboration in pediatric palliative care.*	
**What is New:** • *Besides facilitators and barriers in interpersonal interactions, individual attitudes and perceptions affect interdisciplinary collaboration in pediatric palliative care.* • *Conceptualizing collaboration as situational care networks of non-standard, transmural collaboration between healthcare professionals and parents may strengthen interdisciplinary collaboration.*

**Supplementary Information:**

The online version contains supplementary material available at 10.1007/s00431-026-07070-7.

## Introduction

Interdisciplinary collaboration (IDC) is essential for high-quality pediatric palliative care (PPC) [1–3]. PPC is provided to children with life-limiting conditions [4], among whom an estimated 170,000 die annually within the WHO European Region [4, 5]. While PPC initially focused on symptom treatment during end-of-life, it is now seen as a broader concept representing a holistic, child- and family-centered approach that addresses physical, psychological, social, and spiritual needs to enhance quality of life, dignity in death, and bereavement support [2]. This shift has resulted in more children residing and dying at home [6], giving home-based healthcare professionals (HCPs), next to parents, a significant role in PPC. Yet, specialized expertise remains essential given the rarity and complexity of the medical conditions. Consequently, IDC between multiple HCPs with various professional backgrounds and expertise across home and hospital settings is required to deliver “the right care by the right person at the right place and moment” [2, 4, 7, 8].

IDC is defined as “an effective interpersonal process that facilitates achieving goals that cannot be reached when individual professionals act on their own” [9, 10]. As their child’s legal representative and primary informal caregiver [11], parents are 24/7 deeply involved and thus considered full collaborative partners as well. Important components of IDC are interdependence [9], shared decision-making and goals [9, 12–14], communication and coordination [13, 14], role awareness [15], trust [12, 14], and support [12, 15]. In daily practice, IDC has proven to be challenging [16, 17]. Therefore, multidisciplinary Children’s Palliative Care Teams (CPCTs) have been established to ensure coordination, continuity, and quality of PPC by bridging the gap between hospital and home [18]. Nevertheless, parents report omissions as a result of poor IDC [19, 20]. HCPs, in turn, describe various challenges in collaboration, such as limited contact with other HCPs involved [21].

To understand these challenges, we performed a multiple case study aimed at identifying barriers and facilitators to IDC in PPC, as experienced by expert parents and involved hospital and home-based HCPs.

## Methods

### Design

An exploratory multiple-case study using an inductive thematic analysis was conducted to identify barriers and facilitators for IDC from the parents’ and HCPs’ perspectives [22, 23].

### Sample

A purposive sample of PPC cases was included. Cases consisted of bereaved parents—whose child had passed—and non-bereaved parents—whose child was still living—of primarily home-residing children (0–18 years) with a life-limiting condition and involved HCPs. Maximum variation was sought with respect to diagnosis and illness stage. Therefore, cases of deceased children were also included within 6 months after death, as participants could provide valuable and recent information about all stages, including the terminal stage. HCPs of interest were primary clinicians (mostly a (specialized) pediatrician), general practitioners (GPs), (pediatric) homecare nurses, members of the CPCT, and physiotherapists, as they are key players in PPC. Eligible cases were identified in three of the seven Dutch Pediatric Palliative Care regions [18]. The research team informed and invited parents, after permission to contact them. Following the interview, parents identified involved HCPs. In case of uncertainty regarding the primary clinician, all pediatricians identified were interviewed. If multiple homecare nurses were involved, parents were asked to indicate whom could best elaborate on collaboration.

### Data collection

Semi-structured interviews with parents were conducted at the family’s home. HCPs were individually interviewed via video conference. Topic guides, based on literature and expert knowledge, were largely identical for parents (Supplemental file 1) and HCPs (Supplemental file 2). Topics covered perceptions and experiences regarding IDC in this specific case, including barriers and facilitators. Parents were additionally asked to reflect on collaboration with each individual HCP of interest. Additionally, all participants were invited to elaborate on needs and goals for ideal IDC. The topic guidewas adapted based on insights that relied on iteration of data collection and analysis in the first four cases. Data collection continued until code saturation was reached for the main categories [22, 24]. Descriptive background information was collected by questionnaires.

### Data analysis

Interviews were audio-recorded, transcribed verbatim, verified against the audio records, and pseudonymized. Due to rapid inclusion of cases, data analysis was conducted by two teams with MMK and MCK as linking pin. A multidisciplinary analysis team, consisting of MMK (interviewer, PhD student, physician), JA (researcher, former nurse), MCK (researcher, former pediatric nurse), MdG (nurse specialist CPCT), and ME (researcher, former oncology nurse), was involved in the analysis of the parental interviews and the entire analysis from data towards results, ensuring researcher triangulation [23]. A second analysis team consisting of MMK, MCK, and four medical students analyzed the HCPs interviews.

The inductive thematic analysis was guided by the Qualitative Analysis Guide of Leuven (QUAGOL), which is designed to address heterogeneity in complex, multi-perspective data to enable in-depth understanding of complex phenomena [23]. QUAGOL involves an iterative analytical process that focuses on achieving intersubjective agreement [25], combining systematic coding with collaborative reading and discussion to support within-case and between-case interpretation [23]. The analysis was performed in two phases [23, 25]. First, all team members individually read the interviews to familiarize themselves with the data and to work towards consensus of interpretation. For both parental interviews and HCPs interviews, the outcomes of the analysis teams were then integrated by the multidisciplinary analysis team, where codes and initial categories were discussed and identified, resulting in an initial code tree. In the second phase, new interviews were read and discussed by at least two team members. MMK and the medical students coded all transcripts using NVivo14 software [26], iteratively evaluating and revising the code tree. To ensure validity and transparency, memos were written throughout the analysis, detailing data interpretations, and (relationships between) substantive concepts. Background data of each case were descriptively analyzed using SPSS software, version 29.0 [27].

## Results

### Participants

Within nine cases, representing nine children, 14 parents and 39 HCPs participated in nine interviews and 39 interviews, respectively. Four fathers declined participation because of limited involvement, illness, or work commitments. Eight HCPs declined participation or did not respond. For details, see Table 1. Cases varied in the child’s diagnosis, age, illness stage, and family characteristics such as cultural background. See Table 2 for case and respondent characteristics. Interviews were conducted between September 2023 and April 2024 and lasted 68–133 min (parents) and 21–55 min (HCPs).

**Table 1:** Overview of recruitment and inclusion of participants per case

Case	Mother	Father	Primary clinician	Other clinician	CPCT	GP	Homecare nurse	Physiotherapist
1	1	1	1	1	1	x	1	x
2	1	0 (Occupied (ill))	1	1	1	1	1	1
3	1	1	1	1	x	1	1	1
4	1	1	1	0	1	1	1	1
5	1	0 (Occupied (work))	1	2	1	x	1	1
6	1	1	1	0	1	x	1	x
7	1	1	1	0	1	x	1	x
8	1	0 (Occupied (work))	x	0	1	1	0	1
9	1	0 (Limited involvement)	1	0	1	x	1	1

*CPCT* Children’s Palliative Care Team, *GP* general practitioner*, x* wished not to participate

**Table 2:** Characteristics of the children, parents, and healthcare professionals for each case

	Characteristics		*n* (%)
Child	Gender	Men	5 (56%)
		Women	4 (44%)
	Age at death/at interview	< 1 year	1 (11%)
		1–4 years	3 (33%)
		5–12 years	3 (33%)
		> 12 years	2 (22%)
	Diagnosis	Cardiological	2 (22%)
		Congenital	1 (11%)
		Oncology	1 (11%)
		Metabolic	2 (22%)
		Neurologic	3 (33%)
Parents	Gender	Men	5 (36%)
		Women	9 (64%)
	Age	30–39 years	7 (50%)
		40–49 years	6 (43%)
		> 50 years	1 (7%)
	Marital stage	Married/cohabiting	13 (93%)
		Divorced/not cohabiting	1 (7%)
	Level of education	High school	1 (7%)
		Secondary vocational education	2 (14%)
		Higher vocational education	7 (50%)
		University	4 (29%)
	Religious belief	Christian	6 (43%)
		Muslim	1 (7%)
		None	7 (50%)
Healthcare professionals	Gender	Men	5 (13%)
		Women	34 (87%)
	Age	20–29 years	3 (8%)
		30–39 years	5 (13%)
		40–49 years	9 (23%)
		50–59 years	14 (36%)
		≥ 60 years	8 (21%)
	Vocation	Pediatrician*	13 (33%)
		General practitioner	4 (10%)
		Nurse of the CPCT	8 (21%)
		Pediatric homecare nurse	8 (21%)
		Physiotherapist	6 (15%)
	Years of working experience in PPC	< 5 years	14 (36%)
		5–9 years	8 (21%)
		10–19 years	6 (15%)
		≥ 20 years	11 (28%)
	Number of children qualifying for PPC in care	< 5 per year	16 (41%)
		5–9 per year	5 (13%)
		10–19 per year	6 (15%)
		≥ 20 per year	12 (31%)

^*^General pediatrician (*n* = 3), pediatric oncologist (*n* = 1), pediatric nephrologist (*n* = 1), pediatric neurologist (*n* = 4), pediatric cardiologist (*n* = 2), pediatric metabolic disease specialist (*n* = 1), pediatric revalidation specialist (*n* = 1)

### Barriers and facilitators

We identified facilitators and barriers across seven domains. Whether factors functioned as facilitator, barrier, or both appeared to be context-dependent. Key facilitators and barriers per (sub)domain are presented below, with a full overview and illustrative quotes in Table 3.

**Table 3:** Overview of identified domains, facilitators, barriers, and illustrative quotes

Domain	Subdomain	Facilitator	Barrier	Illustrative quote
Domain 1 Network	1.1 Overview of involved healthcare professionals	Having a good overview of involved healthcare professionals	Lacking an overview of involved healthcare professionals	*“*But gradually you realize that there are so many people involved, and I think, well, there’s actually no good overview of all these people involved. And things keep changing. And you can’t keep up with that either.” – R13 (nurse Children’s Palliative Care Team)
	1.2 Healthcare professionals knowing each other	Knowing each other Natural encounters	Not knowing each other Lack of natural encounters	“And now it’s just been a bit more distant, with an occasional phone call or letter. And everything is probably in there, so there’s nothing to complain about in terms of it not being good or complete, but when you know someone’s face […] then calling each other is easier.” – R51 (general practitioner)
	1.3 Healthcare professionals’ awareness of being a network	Awareness of being a network in this temporary, child-specific situation Positive experiences of working as a network	Unawareness of being a network in this temporary, child-specific situation	“… for years we have been involved, and yet, in that final phase, those first end-of-life discussions, we don’t get invited to participate. Because, again, it all takes place in the hospital. Homecare is almost never… well, let's just say that no one even thinks about the fact that we exist.”* –* R30 (homecare nurse)
Domain 2 Inter-dependence	2.1 Mutual dependence between healthcare professionals	Awareness of mutual dependence Advancing the field	Unawareness of mutual dependence Habitation to reactive care and hesitance to contact other healthcare professionals	“What I learn from this is that we always… we now have enough chronically ill children, that we always insist on involving the general practitioner in the entire process. Even though the pediatrician at the hospital is in charge, you have to involve the general practitioner. Because if we want to provide end-of-life care at home, then you simply need that general practitioner.”- R22 (nurse Children's Palliative Care Team)
	2.2 Mutual dependence between parents and healthcare professionals	Parents feeling dependent on healthcare professionals for support Healthcare professionals feeling dependent of parents’ information	Parents feeling dependent on healthcare professionals for support Dual role of parents Fear of expressing collaborative dissatisfactions	[Ed.: conversation about a moment in the trajectory where parents had expressed their dissatisfaction about a conversation with their primary clinician]: “I indicated that I didn’t mean it that way. But that didn’t feel quite right either, because I thought, yes, it is true, but I have to maintain a good relationship with you. And that makes it a bit dangerous, it makes you very careful about what you do and don’t say.”—R3 (mother)
Domain 3 Goals of care	3.1 Clarity on goals of care	Having clarity on goals of care	Lacking clarity on goals of care	“No, what is difficult with Noah is that his parents kept pushing boundaries. So, Noah doesn’t express anything anymore, he doesn’t smile, he doesn’t cry, he can’t do anything. And when he did, his parents always said that if his quality of life deteriorates and he becomes less comfortable, then maybe we should take a different path. But in fact, they kept extending that period. So now you’re left wondering: is he still comfortable, does he still have quality of life, shouldn’t we talk about that? I find that difficult.” – R31 (homecare nurse)
	3.2 Consensus on goals of care	Having consensus on goals of care Healthcare professionals’ willingness to align with the parents’ goals of care	Lacking consensus on goals of care	[Ed.: conversation about how, in the terminal phase, every healthcare professional focused on “dying well”] “For example, there was a physiotherapist who had been involved long before Amir entered the terminal phase. […] She had a tendency to say that we still needed a patient lift and this and that, so that made things more complicated.” – R46 (homecare nurse)
Domain 4 Roles and tasks	Roles and tasks	Healthcare professionals’ awareness of the need of coordination and agreements on roles and tasks	- Healthcare professionals’ unawareness of the need of coordination and agreements on roles and tasks Domain thinking Healthcare professionals persisting in their habits and perceptions on roles and tasks	“When you talk about involving the Children’s Palliative Care Team, our focus is mainly on end-of-life care. Especially when it comes to coordinating with homecare and such, creating guidelines… So when it becomes more concrete and intensive” – R12 (pediatrician)
Domain 5 Added value	5.1 The added value of interdisciplinary collaboration to child, family, and healthcare professional	Seeing the added value of interdisciplinary collaboration to child, family, or themselves	Not seeing the added value of interdisciplinary collaboration to child, family, or themselves	“At first, I was a little reluctant to involve the Children’s Palliative Care Team. I said, well, I don’t think now is the right time for that. Because there were already so many people involved with this family and this girl that I didn’t see the added value of it. But once the decision was made to discontinue treatment, the expertise of the Children’s Palliative Care Team was incredibly helpful.” – R28 (pediatrician)
	5.2 The personal added value of providing pediatric palliative care	Being able to take a step back	Finding it hard to take a step back when appropriate Fear of losing close involvement and being of meaning	“I would see it as abandoning someone if you say, well, you know, my role as [primary clinician] is now over, because this patient no longer needs [life-prolonging] therapy, so I’m withdrawing. Because I was of course very involved with this girl and her family.”—R28 (pediatrician)
Domain 6 Responsibility	Responsibility	Parental directorship of care Healthcare professionals compensating for shortcomings in interdisciplinary collaboration	Parental directorship of care Healthcare professionals compensating for shortcomings in interdisciplinary collaboration	“Shouldn’t you [involved HCPs] know what each other is doing? Why is it my responsibility? I did take on that role at the beginning and also… as I just mentioned, it gave me a sense of satisfaction. I felt so powerless in the face of epilepsy, but we could do this, coordinate and arrange things. But I don’t have the knowledge and expertise to know who needs to be called in and when. So in the beginning, I felt very lonely in that.”—R11 (mother)
Domain 7 Urgency	7.1 Different perspectives		Different perspectives between parents and healthcare professionals on what is a stable condition or urgent situation	“Yes, because I want her to get better now as well, but, uhm, I don’t feel that urgency as much now, because I think it’s a fairly slow process… …well, it’s pretty calm right now. Or at least, yeah, it’s hard to explain it clearly, but look, she is deteriorating, but she’s deteriorating very slowly. And there, yeah, I don’t feel like we’re not doing things right in terms of care. But then you miss out on the… the need to do something. – R44 (nurse Children’s Palliative Care Team)
	7.2 Feeling an urgency for interdisciplinary collaboration	Healthcare professionals feeling an urgency for interdisciplinary collaboration	Lack of time and competing priorities	“On the one hand, I think it’s very good to coordinate care, but on the other hand, we have a lot of children with multiple disabilities, with really serious problems, and I think it can sometimes be quite a time investment that doesn’t really fit in with the current organization of care.” – R48 (pediatrician)

Some quotes were slightly edited to increase readability. Names are fictitious

#### Domain 1 Network

##### Subdomain 1.1 Overview of involved HCPs

Both parents and HCPs mentioned it was difficult to keep track of the many HCPs from various disciplines involved because of the child’s complex care needs.

Parents mentioned that, although they could develop *a good overview of involved HCPs* based on their involvement in their child’s care, they easily lost the overview in the hectic of caregiving and in times of physical and mental distress. They struggled with whom to approach for specific needs or information.

##### Subdomain 1.2 HCPs knowing each other

*Knowing* *each other*, e.g., by working within the same organization or having spontaneous encounters at the child’s home or in multidisciplinary meetings, facilitated collaboration among different HCPs.

Homecare nurses and GPs emphasized *the lack of natural encounters* with other disciplines and perceived this as a barrier.

##### Subdomain 1.3 HCPs’ awareness of being a network

When involved, HCPs predominantly focused on fulfilling their own tasks. However, many of them described situations, such as medical crises and the terminal stage, during which they developed collaboration as they needed each other’s expertise and efforts to sustain good quality PPC. This activated the *awareness of being a network in this temporary, child-specific situation,* which facilitated IDC, as HCPs actively reached out for each other, attempted to support one another, and organized care together.

Additionally, *positive experiences of working together as a network* increased work satisfaction and, as such, worked as a facilitator.

#### Domain 2 Interdependence

##### Subdomain 2.1 Mutual dependence between HCPs

HCPs varied in the degree of feeling dependent on the input or decisions of other HCPs. HCPs who reported to act relatively independent, such as pediatricians or physiotherapists, relied on their own opinion and decisions in fulfilling their tasks, showing little awareness of the consequences for other HCPs or for their needs. This *unawareness of mutual dependency* hindered IDC, as initiatives to exchange information or to involve others in the decision-making process were not taken.

HCPs who felt dependent on independently operating HCPs, such as GPs or homecare nurses, often did not raise this issue, nor took the initiative to proactively contact the pediatrician. Instead, they waited for others to contact them. This *habituation to reactive care and the hesitance to contact other HCPs* hindered initiatives to start or sustain the collaboration process, acting as a barrier.

Homecare nurses, CPCTs, and pediatricians, who had gained PPC expertise, emphasized the importance of *advancing the field* by contributing to the professional development of less experienced HCPs. Less experienced HCPs highly valued this exchange of expertise, describing it as confidence-building and lowering the threshold to seek support and collaboration, thereby facilitating IDC.

##### Subdomain 2.2 Mutual dependence between parents and HCPs

Both parents and HCPs described to depend on each other to accomplish relevant aims and tasks.

Parents emphasized that HCPs were gatekeepers regarding access to required resources or support. In addition, parents were well aware that HCPs could have different views regarding the care-needs of their child. As such, for the sake of their child, they searched for an alliance that enabled HCPs to empathize with their view as parents. This *feeling dependent on HCPs for support* primarily facilitated IDC, as parents made efforts to stay on speaking terms and avoid tensions with HCPs. However, in the face of child loss, or in the difficulty of deciding what is in the best interest of the child, this could result in negotiations which complicated IDC.

Parents and HCPs often withheld open communication, as they found it difficult to discuss the “frayed edges” of the collaboration process and to communicate about the child’s deterioration coming closer to end-of-life. Parents also mentioned that expressing dissatisfactions about collaboration might lead to HCPs’ disengagement, which could negatively affect their child’s care. This *fear of expressing collaborative dissatisfactions* hindered IDC, as it led to the avoidance of evaluating collaboration.

HCPs, in turn, regarded parents as their most important informants, as parents know their child best and are updated on the most recent developments. HCPs therefore *felt dependent on parents’ information*, which facilitated IDC as HCPs fostered a positive relation with parents as well.

HCPs also struggled with parents not only being collaborative partners but focus of care as well. This *dual role of parents* hindered IDC, as it prompted HCPs to be cautious in open communication on care and collaboration as well.

#### Domain 3 Goals of care

##### Subdomain 3.1 Clarity on goals of care

Parents and HCPs emphasized that *clarity on goals of care* facilitates IDC. It provided them direction in focus and organization of PPC. Knowing the pursued goals of care enhanced parents’ understanding of what to expect from HCPs.

For HCPs, clear goals prevented contradictions, confusion, and uncertainties regarding their own and others’ actions, for instance in the notoriously difficult transition towards comfort care as the main goal.

##### Subdomain 3.2 Consensus on goals of care

Homecare nurses and physiotherapists described that goals of care were often initiated by pediatricians within an academic hospital setting and medically oriented. However, home-based HCPs witnessed whether this policy fitted the child’s situation at home or not. Irritations arose when HCPs in homecare felt forced to follow or contribute to goals they did not (fully) support. This *lack of consensus on goals of care* hindered IDC, as some home-based HCPs pursued their own approach.

Notably, most HCPs were willing to align with parents’ preferred care goals, even without full consensus, believing this would help parents’ coping and grieving process. As this made parents feel taken seriously as collaborative partners, *HCPs’ willingness to align with parents’ care goals* facilitated IDC.

#### Domain 4 Roles and tasks

IDC was hindered when *HCPs persisted in habits and perceptions about roles and tasks*, e.g., pediatricians often only sought collaboration with GPs and CPCTs in later stages, perceiving their roles solely applied to the terminal phase, thereby overlooking their potential roles in earlier phases.

Also, *“domain thinking”*, taking form of limiting IDC activities to the professional domain and/or organization to keep roles and tasks low key, worked as a barrier to IDC as PPC care is characterized by complications and crisis, requiring expertise and flexibility in collaboration.

As the illness progressed, HCPs increasingly became *aware of the necessity of coordination and agreements on roles and tasks*. This facilitated IDC by inducing conversations about what each HCP needed to effectively fulfill their role, providing clarity on roles and tasks, improving mutual understanding and expectations on each role.

#### Domain 5 Added value

##### Subdomain 5.1 The added value of IDC to child, family, and HCP

Parents and HCPs continuously weighed IDC’s benefits for the child, family, and/or themselves against the personal efforts it required. *Not seeing IDC’s added value* hindered IDC, as personal benefits such as time saving, or avoiding collaboration with individuals they did not get along with were likely to be prioritized.

##### Subdomain 5.2 The personal added value of providing PPC

Having longstanding relationships with the families was of added value to HCPs. It was described as meaningful and rewarding by most HCPs. *Fear of losing close involvement and being of meaning* to the family could hinder IDC. For example, some pediatricians feared the CPCT might take over their meaningful relationship with the family. They therefore sometimes postponed the CPCT’s involvement or found it hard to step back when appropriate when care needs changed. Their continued involvement without explicit tasks obstructed optimal IDC between HCPs with active roles and responsibilities.

#### Domain 6 Responsibility

Parents described how their child’s illness made them feel highly responsible for the child’s quality of life. They sought to ensure that PPC was optimally organized and felt compelled to step in if they considered it not fit for their child or once they felt that no one knew their child’s and families’ needs as well as they did. They described putting themselves in intermediate positions between hospital- and home-based HCPs, managing care coordination and information exchange. Although *parental directorship of care* often temporarily solved problems, it sometimes caused friction with HCPs. Homecare nurses and physiotherapists in particular felt as if they had to obtain parents’ permission for interprofessional consultations, disrupting IDC.

Pediatricians, homecare nurses, and physiotherapists described to feel highly responsible for the child’s and family’s wellbeing and the intention to alleviate their burden. When they considered others’ work as insufficient, they tended to take extra steps, take over responsibilities, or bypass other HCPs. Yet, this behavior of *HCPs compensating IDC shortcomings* disrupted the formal IDC process.

#### Domain 7 Urgency

##### Subdomain 7.1 Different perspectives

From a parental view, *different perspectives between parents and HCPs on what was a stable condition or urgent situation* hindered IDC. Parents noted feeling alone after diagnosis, expressing an urgency for support and joint problem-solving, while pediatricians tended to focus on disease and symptom management. They often perceived the situation as stable after diagnosis, deprioritizing interdisciplinary information exchange and care coordination.

##### Subdomain 7.2 Feeling an urgency for IDC

An important facilitator surfaced when HCPs *felt the urgency* for IDC, which was typically in times of crisis or the terminal phase, as they realized they had no other choice but to address issues collaboratively. Driven by this urge and the feeling of having one chance to do it right for this family, pediatricians, GPs, homecare nurses, and CPCTs described how they organized IDC, e.g. by organizing multidisciplinary team meetings and coordinating roles and care goals and high-frequency communication via secured clinical messaging platforms.

#### Synthesis

HCPs took the initiative to arrange or intensify IDC when complex situations arose, such as medical crisis or far-reaching decision-making, because then the urgency and the added value of collaboration were felt. In particular at the end-of-life, collaboration intensified. Not only because of medical complexity, but also because HCPs felt responsible for good care and parental support during end-of-life, as this could be done only once. In contrast, parents lacked this, taking their perspective after diagnosis. With HCP’s decreased collaboration efforts because of the decrease in medical issues, parents expressed a need for continuation of collaboration to help them with their new responsibilities as parental caregivers and in the coordination of the increasing number of HCPs that became involved. This tension between perceived medical stability from HCPs’ perspectives and parents’ lived experience of living with illness hindered IDC systematically. Once exerting IDC, many respondents experienced its added value for themselves and other stakeholders and emphasized that this should be arranged earlier in the illness trajectory.

## Discussion

In this exploratory multiple-case study, facilitators and barriers to IDC in PPC as perceived by parents and HCPs were examined. Factors acted as facilitators, barriers, or both and were categorized into seven domains: network, interdependence, goals of care, roles and tasks, added value, responsibility, and urgency. A few results stood out. IDC intensified during crises and in the terminal phase when awareness of being part of a network and the recognition of IDC’s need and added value was highest. Parents often felt compelled to compensate for poor collaboration to meet their child’s needs, as IDC developed slowly after diagnosis due to barriers such as HCPs’ focus on medical complexity, competing priorities and hesitation to reach out for colleagues in other disciplines and settings.

Our findings on identified facilitators and barriers are in line with existing literature, e.g., (un)awareness of interdependence [9], (lack of) clarity and consensus on care goals [9], (not) discussing role distributions [9, 16, 17], and (a lack of) communication [16, 17]. Yet, the finding that many barriers are related to the individual HCP’s perceptions and attitudes regarding collaboration and the personal tradeoff between efforts and benefits is hardly exposed in existing literature. It can be concluded that the advancement of IDC in PPC does not come about by itself nor can it rely on the individual HCPs’ perceptions of its usefulness or their willingness and capacity to prioritize collaboration and the family’s needs in busy day-to-day practice [28]. To mitigate the impact of negative attitudes and perceptions, and to prevent these from outweighing initiatives to collaborate, IDC should be framed as a routine and integral component of caring for child and family. Consistent with the CanMEDS [29] framework, collaboration should be recognized as a core professional competence and structurally embedded and evaluated in daily practice by assigning the responsibility for IDC as a formal task to a key professional, e.g., a case manager [30]. This includes an early and ongoing dialogue between involved HCPs and between HCPs and parents to ensure that perspectives, needs, and desired support are made explicit, which is in line with NICE guidelines [8].

This study highlights that IDC between parents and HCPs is not easily established. While HCPs primarily focus on symptom management [19, 31], parents adopt a more holistic focus on quality of life. As such, parents feel responsible to compensate for gaps in coordination. This undermines their ability to “just be parents” [32, 33] and increases their workload [31, 34]. However, parental directorship is also highly important to parents as a form of control in an uncertain situation. For HCPs, this raises the dilemma in balancing care delivery for parents, who are both focus of care and collaborative partners at the same time. Open communication and integration of parental perspective is therefore required, although parents may be reluctant to express dissatisfaction due to fear of triggering HCP disengagement. This highlights the need for HCPs to proactively seek feedback and foster trust and to separate care provision from the provision of feedback. This separation can be supported by an independent intermediary, such as a case manager, who can facilitate coordination and feedback. Equally important is that parents experience HCPs’ presence, rather than being positioned opposite them, which represents a holistic approach to PPC. Yet, as this is a relatively new but important finding, further research should explore how open communication between parents and HCPs can be supported.

HCPs mentioned that care situations differed per child, but all improved through collaboration with other HCPs. Such situational collaborations can be conceptualized as *situational care network* (SCN*)*: a temporary network comprising parents and a fluctuating number of often nonstandard collaborating HCPs, who are involved for a specific period in the disease trajectory of an individual child and therefore have to collaborate. Importantly, the concept of SCNs offers a frame for complex medical situations involving nonstandard collaborating HCPs, throughout an illness trajectory. The concept of a SCN supports high-quality, coordinated and continuous care by acknowledging challenges such as limited partner visibility [35, 36], and fewer natural opportunities for communication [35, 37] and familiarization [16]. Simultaneously, SCNs facilitate IDC in PPC, including mutual support [16], shared learning [16], resource access [16], and complementary roles [38] and expertise. Triggers for SCN meetings to align IDC should be embedded throughout the PPC trajectory, including diagnosis or ongoing diagnostic uncertainty, crisis, clinical deterioration, and the terminal phase. Meetings are also warranted when changes occur within the network, such as shifts in composition, or in case of physical or developmental transitions of the child, e.g., complicated transfers due to the child’s increasing weight or the 18 −/18 + transition. Finally, SCNs should respond to parental needs, such as caregiver overload or reduced capacity to coordinate care. Further implementation-oriented research is needed to operationalize and evaluate these triggers in practice.

To the best of our knowledge, this is the first multiple-case study that, in a large sample, systematically provided insights into parents’ and various HCPs’ perspectives within the same cases and across all phases of the PPC trajectory.

A few limitations should be noted. First, the parents in this sample were highly educated (79% with higher vocational or university education), and both parents and HCPs highlighted involved parents had good coordination skills. Yet, HCPs cautioned that such capabilities cannot be assumed for all families. Rather than interpreting these parents’ capacities as standard, this underscores the need for an ongoing dialogue with families about their support needed. Moreover, a well-functioning, collaborative SCN is essential to ensure coordinated, tailored PPC and reduce the burden on parents to coordinate collaboration. For families with fewer resources or limited capacities to take on coordinating roles, SCNs should adapt by explicitly discussing and redistributing responsibilities within the network, for example, by appointing a case manager or key professional to (partly) assume coordination tasks.

Second, four fathers declined participation, which may introduce bias, particularly in Domain 6 (Responsibility), given that gendered caregiving roles can shape perceptions of coordination burden. Interviews, especially those with both parents, highlighted that mothers indeed assumed primary responsibility for care coordination. This may suggest a pattern in which mothers feel higher senses of responsibility resulting in more coordination burden. However, this should be interpreted with caution, as not all fathers’ perspectives were captured, limiting firm conclusions about the distribution of coordination roles and responsibility burdens between parents.

In conclusion, this multiple-case study identified facilitators and barriers for IDC in PPC in the domain network, interdependence, goals of care, roles and tasks, added value, responsibility, and urgency. To strengthen IDC in PPC, a first step is to conceptualize collaboration as occurring in *situational care networks*. Early recognition of a *situational care network* seems to strengthen structural IDC in PPC, rather than reactive reliance on parental directorship or crisis-driven senses.

## Supplementary Information

Below is the link to the electronic supplementary material.

ESM 1 (21.0 KB DOCX)

ESM 2 (20.2 KB DOCX)

## Data Availability

The data are available upon request.

## Abbreviations

CPCTs: Children’s Palliative Care Teams
HCPs: Healthcare professionals
IDC: Interdisciplinary collaboration
PPC: Pediatric palliative care
SCN: Situational care network
