# Supplementary material for: Interdisciplinary collaboration in pediatric palliative care: a qualitative study on barriers and facilitators as perceived by parents and healthcare professionals
Source: Eur J Pediatr. 2026 May 20;185(6):419. doi: 10.1007/s00431-026-07070-7 (PMC13190755; doi:10.1007/s00431-026-07070-7)
Supplement: Supplementary file 1 — (21.0 KB DOCX) [file 431_2026_7070_MOESM1_ESM.docx]

**Interdisciplinary collaboration in pediatric palliative care: a qualitative study on barriers and facilitators as perceived by parents and healthcare professionals**

**Topic guide parents***The same topic guide was used for bereaved parents; only questions referring to actual care for the child were asked in the past tense.*

Introduction question

This conversation is mainly about [name child] and the care for him/her. I am not familiar with you or your family, so I would appreciate getting to know you and your child a little. Could you tell me something about who [name child] is and what kind of condition he/she has?

Opening question

Caring for children with a serious illness such as [name child] requires expertise and a patient tailored approach. This usually means that, besides you as his/her parent, multiple healthcare professionals are involved. With this interview, I would like to understand how you experience the collaboration with healthcare professionals, and how you perceive the mutual collaboration between involved professionals. What goes well, what can be improved, and in what way?

Topic guide (key questions)

| **Theme** | **Research question** | **Interview question*** | **Optional additional questions*** |
| --- | --- | --- | --- |
| Network | How do parents perceive their relationship with the respective healthcare professionals?  (general practitioner, homecare nurse, primary clinician, children’s palliative care team, physiotherapist)  According to parents, what role does the respective healthcare professional have within the network surrounding the serious ill child and the family?  Do parents have insight into interdisciplinary collaboration between the different healthcare professionals? | On a scale of 1-10, what rating would you give this healthcare professional?  (general practitioner, homecare nurse, primary clinician, children’s palliative care team, physiotherapist)  What task or responsibility does he/she take in the care for [name child]?  Do you notice any collaboration between the healthcare professionals? | - What makes you give this rating? E.g. trust, expertise, long-standing relationship, taking responsibility, equality  - What does he/she do well?  - If you have to be critical and mention a suggestion for improvement, what would that be?  - How does he/she perform this task?  - How is that for you?  If yes:  - Can you give an example?  - How do you notice this?  - How does that go?  If no: - What do you think about that?  - How do you think this should be done?  - Is it clear to you who to contact in case of problems?  If collaboration occurs via parents:  - Is there a specific reason for that?  - Is it more about informative consultation rather than policy-related consultation? |
| Collaboration in pediatric palliative care | Which factors play a role for parents in positively experienced situations regarding interdisciplinary collaboration in the care for their serious ill child?  What challenges do parents experience in interdisciplinary collaboration in the care for their serious ill child? | Can you give an example of a situation where you experienced good collaboration between the involved healthcare professionals?  What does not get off well or stagnate in the joint care for [name child]? | - What makes it good?  - Who plays an important role in this?  - Does it result in good care?  - What lessons could be drawn from this for the future?  - What causes this?  - Does collaboration play a role in this?  - Is this discussed?  - What should we learn from this for the future? |
| Interdisciplinary collaboration | Do parents feel part of the interdisciplinary team involved in the care of their child?  Is the current form of interdisciplinary collaboration in the care for their child proactive, according to the parents?  How does the ideal situation of interdisciplinary collaboration in the care for serious ill children look like, according to parents? | When we talk about care networks around a child, what is your role as parents in such a network?  When a problem arises in the care for [name child], how is this managed?  Imagine you live in an ideal world where you can decide how care should be organized, how would the care for [name child] look like? | - Do you feel taken seriously?  - Do healthcare professionals also see it that way?  - Do you and the healthcare professional share the same goals regarding care?  - Can you give an example? How was it resolved? Or is it not resolved?  - What impact does that have on you?  - Does an individual play a role in this?  - Does collaboration play a role in this?  - What should remain the same? With whom? Why?  - What should be changed? With whom? Why?  - Do you see opportunities for that? Why (not)? |

Ending question

Are there any issues you were not able to fully express or that you would like to mention?

Closing

Thank you for allowing me to interview you and for sharing your experiences with me.

* Prompts:

- Can you give an example of that?
- Can you tell me more about that?
- How did that work in practice?
- What caused that …?
- Can you describe that for me?
- I do not fully understand; could you explain that again?
- How did that continue?
- What did that mean for you?
- What did you think of that?
