# Supplementary material for: Interdisciplinary collaboration in pediatric palliative care: a qualitative study on barriers and facilitators as perceived by parents and healthcare professionals
Source: Eur J Pediatr. 2026 May 20;185(6):419. doi: 10.1007/s00431-026-07070-7 (PMC13190755; doi:10.1007/s00431-026-07070-7)
Supplement: Supplementary file 2 — (20.2 KB DOCX) [file 431_2026_7070_MOESM2_ESM.docx]

**Interdisciplinary collaboration in pediatric palliative care: a qualitative study on barriers and facilitators as perceived by parents and healthcare professionals**

**Topic guide healthcare professionals***The same topic guide was used for healthcare professionals involved in care for a deceased child; only questions referring to actual care for the child were asked in the past tense.*

Opening question

This interview is about the case of [name child] and the care for him/her. Could you first describe how you became involved in the care for [name child]?

Introduction question

Palliative care for children is not easy and requires a tailored approach. As a result, multiple healthcare professionals are often involved, which requires collaboration. Collaboration can be complex, both with parents and with other professionals. Through this interview, I aim to gain insight into what challenges you experience in this, what areas for improvement you see, and if so, in what way.

Opening question

This interview is about the case of [name child] and the care for him/her. Could you first describe how you became involved in the care for [name child]?

Topic guide (key questions)

| **Theme** | **Research question** | **Interview question*** | **Optional additional questions*** |
| --- | --- | --- | --- |
| Network | How does a healthcare professional define interdisciplinary collaboration?  What role does the healthcare professional have in caring for a child qualifying for pediatric palliative care?  Does the healthcare professional have insight into and contact with all other healthcare professionals involved in care for this child and family? | When talking about interdisciplinary collaboration, what does that mean to you?  What do you see as your role or responsibility in the care for [name child]?  Do you have contact with other healthcare professionals regarding the care for [name child]? | - Do others see it that way as well?  - Are there any misconceptions about your role? By whom?  - Are there any changes in your role during the palliative trajectory? How?  - How does the contact with other healthcare professionals go? And with the parents?  - Which tasks do others take one?  - Is it clear to you whom to approach in case of problems or concerns?  - Whom do the parents turn to when problems arise? Does this have consequences for others in the network? |
| Collaboration in pediatric palliative care | Which factors positively contribute to interdisciplinary collaboration in pediatric palliative care from the healthcare professional’s perspective?  Which factors create friction in the interdisciplinary collaboration in pediatric palliative care from the healthcare professional’s perspective? | Can you describe a moment in the care for [name child] when collaboration went well?  What does not get off well or stagnate in the joint care for [name child]?  What would you like to discuss with other healthcare professionals? Why does that not happen? | - What makes it good?  - Does it result in good care?  - What is your contribution to this?  - What are the contributions of others?  - What lessons could be drawn from this for the future?  - How does that happen? What is missing?  - What are the contributions of others?  - What lessons could be taken from this for the future? |
| Interdisciplinary collaboration | What is the healthcare professional’s perception of the approach to interdisciplinary collaboration in this case?  How does the healthcare professional ideally envision interdisciplinary collaboration in this case? | Can you describe what providing care for [name child] together with other healthcare professionals looks like?  Imagine you live in an ideal world where you could decide everything, what would providing care for a child like [name child] together with other healthcare professionals look like? | - What goes well?  - What can be improved?  - Are there things you feel are missing? E.g. other professionals, organizational aspects, training?  - Are care goals being discussed and established jointly?  - Is it discussed who is needed and to what extent?  - Do you discuss with others how the care and collaboration are going?  - What should remain the same? With whom? Why?  - What should be changed? With whom? Why?  - Do you see opportunities for that? Why (not)? |

Ending question

Are there any issues you were not able to fully express or that you would like to mention?

Closing

Thank you for allowing me to interview you and for sharing your experiences with me.

*Prompts:

- Can you give an example of that?
- Can you tell me more about that?
- How did that work in practice?
- What caused that …?
- Can you describe that for me?
- I do not fully understand; could you explain that again?
- How did that continue?
- What did that mean for you?
- What did you think of that?
